# Variability in the perception of palliative care and end-of-life care among hematology professionals from the same reference center in Bahia, Brazil: A descriptive cross-sectional study

**DOI:** 10.1590/1516-3180.2023.0255.R1.29112023

**Published:** 2024-02-23

**Authors:** Diego Lopes Paim Miranda, Alini Maria Orathes Ponte Silva, David Pereira Ferreira, Laís Teixeira da Silva, Liliane Lins-Kusterer, Edvan de Queiroz Crusoé, Marianna Batista Vieira Lima, Marco Aurélio Salvino

**Affiliations:** I MD. MSc student, Postgraduate Program in Medicine and Health, Professor Edgard Santos University Hospital, Medical School, Universidade Federal da Bahia (UFBA), Salvador (BA), Brazil.; II MD. MSc student, Postgraduate Program in Medicine and Health, Professor Edgard Santos University Hospital, Medical School, Universidade Federal da Bahia (UFBA), Salvador (BA), Brazil.; III Medicine Student, Medical School, Universidade Federal da Bahia (UFBA), Salvador (BA), Brazil.; IV MD. MSc student, Postgraduate Program in Medicine and Health, Professor Edgard Santos University Hospital, Medical School, Universidade Federal da Bahia (UFBA), Salvador (BA), Brazil.; V PhD. Dental Surgeon, Professor, Postgraduate Program in Medicine and Health, Department of Preventive and Social Medicine Medical School, Universidade Federal da Bahia (UFBA), Salvador (BA), Brazil.; VI MD, PhD. Hospital Universitário Professor Edgard Santos (HUPES), Universidade Federal da Bahia (UFBA), Salvador (BA), Brazil.; VII MD. Physician, Hospital Universitário Professor Edgard Santos (HUPES), Universidade Federal da Bahia (UFBA), Salvador (BA), Brazil.; VIII MD, PhD. Associate Professor, Postgraduate Program in Medicine and Health, Professor Edgard Santos University Hospital, Medical School, Universidade Federal da Bahia (UFBA), Salvador (BA), Brazil.

**Keywords:** Palliative care, Palliative medicine, Hematology, Perception, End-of-life care, Oncohematology, Variability

## Abstract

**BACKGROUND:**

There are several illness-specific cultural and system-based barriers to palliative care (PC) integration and end-of-life (EOL) care in the field of oncohematology.

**OBJECTIVES:**

This study aimed to investigate the variability in the perceptions of PC and EOL care.

**DESIGN AND SETTING:**

A cross-sectional study was conducted in the Hematology Division of our University Hospital in Salvador, Bahia, Brazil.

**METHODS:**

Twenty physicians responded to a sociodemographic questionnaire and an adaptation of clinical questionnaires used in previous studies from October to December 2022.

**RESULTS:**

The median age of the participants was 44 years, 80% of the participants identified as female, and 75% were hematologists. Participants faced a hypothetical scenario involving the treatment of a 65-year-old female with a poor prognosis acute myeloid leukemia refractory to first-line treatment. Sixty percent of the participants chose to follow other chemotherapy regimens, whereas 40% opted for PC. Next, participants considered case salvage for the patient who developed septic shock following chemotherapy and were prompted to choose their most probable conduct, and the conduct they thought would be better for the patient. Even though participants were from the same center, we found a divergence from the most probable conduct among 40% of the participants, which was due to personal convictions, legal aspects, and other physicians’ reactions.

**CONCLUSIONS:**

We found considerable differences in the perception of PC and EOL care among professionals, despite following the same protocols. The study also demonstrated variations between healthcare professionals’ beliefs and practices and persistent historical tendencies to prioritize aggressive interventions.

## INTRODUCTION

A comprehensive and multidisciplinary approach is crucial in oncohematology. Oncohematologic patients often experience higher symptom burden, increased in-hospital mortality, and higher rates of complex care needs during the cancer-associated end-of-life (EOL) process.^1-3^ Palliative care (PC) is uncommon in oncohematology. Although continuous combined care by an interdisciplinary provider team including physicians well-equipped for PC is effective, it is not usually considered.^1,4,5^ Furthermore, limited research has been conducted to explore determinants of the variability in EOL care among healthcare professionals, especially in oncohematology practice.^6,7^

Hematologic malignancies consist of a heterogeneous group of diseases characterized by distinct patterns of illness progression, approaches to treatment and prospects for remission, impacting patients’ requirements for PC and EOL support.^8-10^ The “rollercoaster” nature of life experienced by individuals with these illnesses bring distinct physical and psychological challenges encompassing emotional pain and a multitude of symptoms.^11-14^ Particularly in diseases such as acute myeloid leukemias, patients commonly have a high risk of serious complications and negative outcomes along with high possibility for positive outcomes.^11-14^

EOL is the final phase of patients’ life when a cure is no longer possible, and the focus of care shifts towards providing comfort, symptom relief, and emotional support for patients and families.^8-10^ Several barriers to PC integration and optimal EOL care exist among oncohematologic patients including illness-specificity, cultural differences, and system-based inhibitions.^15-17^ Adequate PC requires appropriate knowledge from the healthcare team, but frequently there are divergences in practice within the same staff, which may hinder the integration and efficiency of patients’ clinical course.^1,3,15^

On a larger scale, PC plays an essential role in coordinated patient care, with the primary goal of promoting relief from physical, psychological, and spiritual suffering while providing family support throughout the disease course.^15,18^ It is extremely important to consider combined care (PC associated with disease-modifying treatment) to enhance the quality of life and symptom control.^5,15,18^

Even among centers with experienced healthcare professionals and specialized PC teams, significant disparities exist in the clinical management and decision-making process.^4,19,20^ A patient-centered approach aims to provide comfort and quality of life regardless of the possibility of a cure.^15,18^ However, in the oncohematology field, there is a historical tendency to prioritize aggressive and curative interventions, often delaying PC strategies.^5,15-17^ Therefore, a major challenge is finding a therapeutic balance to avoid overtreatment while bypassing premature treatment discontinuation that could lead to potentially avoidable deaths.^21,22^

Various reasons have been described as to why physicians tend not to promote EOL care discussions, for example, the perception of weakening the physician-patient relationship due to the restriction of curative measures, the loss of professional credibility, and the feeling of diminishing hope for the patient and family members.^5,23,24^Moreover, this process is often also dependent on what professionals believe is best for the patient based on their personal convictions about healthcare, PC, and EOL care, previous experiences, and cultural aspects.^5,23,24^

Thus, a growing awareness of the importance of combined care among oncohematologic patients has led to critical reflection on the conduct of healthcare professionals.^25,26^ The involvement of the PC team in severe and refractory cases is especially relevant, considering that therapeutic options could generate significant effects that impact patients quality of life.^25,26^ In such circumstances, it is necessary to investigate not only the concordance of conduct within the same healthcare team involved in the care of these patients, but also the presence of determinants of the variability in the establishment of PC and EOL care strategies.^27^

## OBJECTIVE

This study aimed to investigate variability in the perception of PC and EOL care among 20 hematologists and hematology residents from the Hematology Division of the Universidade Federal da Bahia (UFBA) University Hospital.

## METHODS

This cross-sectional web-based survey study was approved by the Ethics Committee of the Medical School of Universidade Federal da Bahia (UFBA) on August 8, 2022 (CAAE 61154522.0.0000.0049) and was conducted according to the Declaration of Helsinki and Good Clinical Practice guidelines (December 2, 2021, number: 12/155).

The hematology service of UFBA’s University Hospital has been consolidated as the only hematology reference center in the state of Bahia, Brazil since the 1990s. This service includes annual curricular activities for three medical residency programs and dozens of medical school students. Since 2010, over 400 oncological treatments have been performed monthly at the University Hospital, and almost 50 bone marrow transplantations have been performed annually.

Fifteen hematologists and five hematology residents (four second-year residents and one first-year resident) from UFBA University Hospital were recruited from October 2022 to December 2022. We recruited 20 hematology team members who provided written informed consent and agreed to participate in the study. Participants were provided with a Google Forms electronic survey through a link sent to them via e-mail or WhatsApp Messenger. A reminder was sent to those who did not respond to the initial invitation to participate.

The questionnaire contained 28 questions, with an approximate completion time of 15 minutes. To submit the questionnaire, each participant was required to log into a Google account and submissions were limited to one per account. The questionnaire consisted of two sections that addressed the sociodemographic and clinical factors.

The first section addressed participants’ personal, professional, and EOL educational characteristics. The participants were analyzed by age, sex, years since graduation in medical school, function in the service, work hours/week at the University Hospital, training in other medical specialties, whether or not participants had had PC and/or EOL classes during their medical education, whether or not they had had law or ethical classes regarding PC and/or EOL during their medical education, PC and/or EOL-themed articles read in the last two years, PC and/or EOL-themed events participated in the last two years, whether or not they had interest in discussing PC and/or EOL, self-attributed knowledge on PC and/or EOL, religiosity, belief in God, and the presence of children.

The second section adapted a questionnaire from a similar previously published study.^6^ It consisted of two fictitious clinical cases constructed by the authors, followed by questions about prognosis and therapeutic possibilities, and the reasons that led the participants to make those decisions. A multidisciplinary team of six members, including palliative and support Care experts, hematologists, and nurses, reviewed the content of the survey to ensure interpretability and applicability to the hematology setting. Four rounds of discussion were necessary to reach a consensus. The questionnaire was written in Portuguese, the official language of Brazil.

The case scenario presented in the second section described a 65-year-old female patient with a poor prognosis of acute myeloid leukemia refractory to first-line treatment. We analyzed the hypothetical conduct in the face of this scenario and two main elements of EOL care: Q1) whether the decision-making process was conducted using a multi-professional approach and Q2) what approach to life-sustaining therapies would be taken if the patient developed septic shock following chemotherapy. In the second question, two additional sub-options were prompted for selection: Q2a) the care clinicians are most likely to deliver, and Q2b) the care they think is best for the patient, given the exact same alternatives to choose from. If the participants selected different answers for 2a and 2b, they were further prompted to explain their reasoning by choosing one reason from a limited number of options.

The data were combined into a common database to ensure the coding and analysis procedures. Data normality was assessed using Kolmogorov-Smirnov and Shapiro-Wilk tests, descriptive statistical measures, and graphic analysis. Descriptive analyses included frequencies and percentages for categorical variables and means and standard deviations or medians and interquartile ranges for continuous variables. Because the study was a census, only descriptive statistics were necessary, considering that statistical inference to estimate population values from samples is trivial in this type of study. The Wilson score method without continuity correction was used to calculate the confidence interval for a proportion.^28^ Statistical analyses were performed using SPSS 25.0 for Windows (Chicago, IL, USA).

## RESULTS

All professionals completed the questionnaire during the study period and consolidated it as a census. Eight physicians completed the questionnaire using hospital facilities in a private room at the University Hospital. They submitted the questionnaire anonymously and the remaining 12 completed it using private mobile devices. The primary characteristics of the 20 physicians are listed in Table 1.

**Table 1.** General characteristics of 20 respondents from the Hematology Division

Characteristics	Results
Age (years)^*^	44 (12)
Female sex	16 (80%)
Years since graduation †	16 (7-27)
Graduated hematologist	15 (75%)
Work hours/week in the University Hospital †	24 (24-54)
Another specialty	8 (40%)
PC and/or EOL classes	14 (70%)
Law and/or ethic classes on PC or EOL	14 (70%)
PC and/or EOL articles	
2-3 articles read/2 years	6 (30%)
4 or more articles read/2 years	8 (40%)
PC and/or EOL themed events	
2-3 articles events/2 years	5 (25%)
4 or more events/2 years	10 (50%)
Interest in discussing PC and/or EOL	20 (100%)
Self-attributed knowledge	
Regular	12 (60%)
Good	6 (30%)
Religiosity	9 (45%)
Belief in God	17 (85%)
Has children	10 (50%)

^*^mean (SD); † median (IQR); PC = palliative care; EOL = end of life; SD = standard deviation; IQR = interquartile range.

Regarding hypothetical conduct in the face of the fictitious scenario, 12 participants (60%; 95%CI = 38% to 78%) chose to follow other chemotherapy regimens, while eight (40%; 95%CI = 21% to 61%) chose to implement PC, with or without transfusion support. A descriptive analysis of the characteristics of the two groups divided by chosen attitudes is shown in Table 2.

**Table 2.** Descriptive analysis of the initial approach to a 65-year-old female patient with poor prognosis acute myeloid leukemia refractory to first line treatment according to physicians’ characteristics

Characteristics	Chosen attitude			
	Other chemotherapy regimen (n = 12)	(95%CI)^a^	Palliative care (n = 8)	(95%CI)^a^
Age (years)^*^	42 (11)	(35-49)	46 (13)	(35-57)
Female sex	8 (67%)	(39% - 86%)	8 (100%)	(67% - 100%)
Years since graduation ^†^	15 (7-24)	(10-24)	20 (8-32)	(6-34)
Graduated hematologist	9 (75%)	(46% - 91%)	6 (75%)	(41% - 93%)
Work hours/week in the University Hospital ^†^	25 (24-54)	(24-60)	24 (24-52)	(24-70)
PC and/or EOL classes	8 (67%)	(39% - 86%)	6 (75%)	(41% - 93%)
Law or ethic classes on PC and/or EOL	8 (67%)	(39% - 86%)	6 (75%)	(41% - 93%)
PC and/or EOL articles				
4 or more articles read/2 years	5 (42%)	(19% - 68%)	3 (37%)	(13% - 69%)
PC and/or EOL themed events				
4 or more events/2 years	6 (50%)	(25% - 74%)	4 (50%)	(21% - 78%)
Interest in discussing PC and/or EOL	12 (100%)	(75% - 100%)	8 (100%)	(67% - 100%)
Self-attributed knowledge				
Good	2 (17%)	(4% - 44%)	4 (50%)	(21% - 78%)
Religiosity	5 (42%)	(19% - 68%)	4 (50%)	(21% - 78%)
Belief in God	10 (83%)	(55% - 95%)	7 (87%)	(52% - 97%)
Has children	6 (50%)	(25% - 74%)	4 (50%)	(21% - 78%)

^*^mean (SD);

^†^median (IQR);

^a^ Wilson score method without continuity correction was used to calculate the confidence interval for a proportion.

PC = palliative care; EOL = end of life; SD = standard deviation; IQR = interquartile range.

All 20 participants (100%) said they would discuss the care pathway with the patient and the multidisciplinary team (e.g., physicians, nurses, psychologists), but 18 (90%; 95%CI = 69% to 97%) said they would predominantly take into consideration the opinion of the patient when making decisions, while only two (10%; 95%CI = 2% to 30%) said they would predominantly take into account the opinion of the multidisciplinary team.

Participants were further asked to consider their conduct when a next-step chemotherapy regimen was adopted, and the patient developed febrile neutropenia, which progressed to septic shock, low peripheral oxygen saturation levels, and a low level of consciousness. Thirteen physicians (65%; 95%CI = 43% to 81%) chose to apply the full code (referral to the intensive care unit, antibiotic treatment, and invasive supportive measures such as cardiopulmonary resuscitation, orotracheal intubation, and hemodialysis, if necessary). However, 7 participants (35%; 95%CI = 18% to 56%) chose to withhold or withdraw treatment interventions. A descriptive analysis of the characteristics of the two groups divided by chosen attitudes is shown in Table 3.

**Table 3.** Descriptive analysis of the most likely approach for a 65-year-old female patient with poor prognosis acute myeloid leukemia refractory to first line treatment, experiencing septic shock following a next step chemotherapy regimen according to physicians’ characteristics

Characteristics	Chosen attitude			
	Full code (n = 13)	(95%CI)^a^	Withholding/ withdrawal (n = 7)	(95%CI)^a^
Age (years) ^*^	41 (11)	(34-47)	49 (13)	(37-61)
Female sex	10 (77%)	(49% - 91%)	6 (86%)	(48% - 97%)
Years since graduation^†^	15 (5-23)	(10-24)	23 (13-38)	(13-44)
Graduated hematologist	9 (69%)	(42% - 87%)	6 (86%)	(48% - 97%)
Work hours/week in the University Hospital^†^	24 (24-60)	(24-60)	24 (24-36)	(24-70)
PC and/or EOL classes	8 (61%)	(35% - 82%)	6 (86%)	(48% - 97%)
Law or ethic classes on PC and/or EOL	8 (61%)	(35% - 82%)	6 (86%)	(48% - 97%)
PC and/or EOL articles				
4 or more articles read/2 years	6 (46%)	(23% - 70%)	2 (29%)	(8% - 64%)
PC and/or EOL themed events				
4 or more events/2 years	8 (61%)	(35% - 82%)	2 (29%)	(8% - 64%)
Interest in discussing PC and/or EOL	13 (100%)	(77% - 100%)	7 (100%)	(64% - 100%)
Self-attributed knowledge				
Good	5 (38%)	(17% - 64%)	1 (14%)	(2% - 51%)
Religiosity	6 (46%)	(23% - 70%)	3 (43%)	(15% - 74%)
Belief in God	12 (92%)	(66% - 98%)	5 (71%)	(35% - 91%)
Has children	5 (38%)	(17% - 64%)	5 (71%)	(35% - 91%)

^*^mean (SD)

^†^median (IQR);

^a^ Wilson score method without continuity correction was used to calculate the confidence interval for a proportion.

PC = palliative care; EOL = end of life; SD = Standard deviation; IQR = Interquartile range.

**Figure 1.**
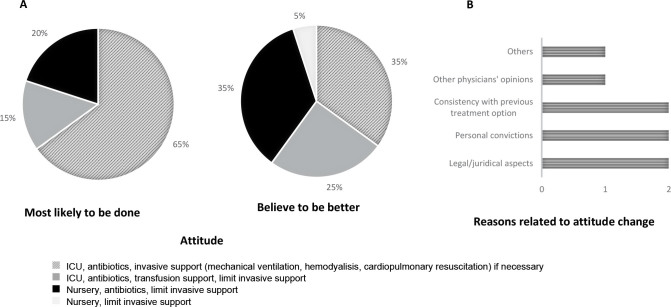
Most likely to be done and believed to be the best treatment for a 65-year-old female patient with poor prognosis acute myeloid leukemia refractory to first line treatment, experiencing septic shock following a next step chemotherapy regimen. **A** Shows the approach most likely to be done and the approach believed to be best for the patient. **B** Shows the reasons related to attitude change in approach of the 8 physicians (40%) that changed answers between the two questions. ‘‘Others’’ denotes one physician who answered that “it is hard to give up”.

When considering the next step of chemotherapy, 8 of the 20 respondents (40%; 95%CI = 21% to 61%) had disagreeing answers about the most likely treatment to be given and the believed to be the best approach for patients experiencing septic shock (Figure 1A). All eight participants (100%) believed that it would be better for the patient to limit invasive supportive measures and strategies than they would choose. The number of participants who chose to apply the full code decreased from 13 (65%; 95%CI = 43% to 81%) to 7 (35%; 95%CI = 18% to 56%) comparing these two possibilities (Figure 1A). The reasons for the differences in these approaches are shown in Figure 1B.

## DISCUSSION

PC in the oncohematology team is particularly pertinent when dealing with severe and refractory cases, as treatment interventions may profoundly affect patients’ overall quality of life.^25,26^ Furthermore, only a limited number of studies have investigated the impact of physicians’ characteristics and their respective approaches in clinical practice.^7,29,30^ Given that the research done on PC in an oncohematological clinical setting is scarce, our study is crucial and makes a significant contribution to a clinical environment that demands renewed analyses and heightened attention. This study will prompt clinicians to contemplate the underlying paradigms and representations that shape their professional interventions. This introspection is vital to facilitate the initiation of EOL discussions and promote the early integration of PC practices.

In our hypothetical case study, most participants opted to pursue alternative chemotherapy regimens, and a minority opted to pursue PC interventions with or without transfusion support. This highlights the prevailing inclination to explore non-PC options, aligning with the existing literature on this subject.^16,31^ We also found a small disparity in the number of years since graduation between those who opted for PC and those who chose alternative chemotherapy regimens. The former group had completed a median of 20 years (interquartile range 8-32) since graduation, whereas the latter group had completed a median of 15 years (interquartile range 7-24). This discrepancy suggests a tendency among more experienced professionals to lean towards PC as an approach.

Furthermore, it is crucial to emphasize the percentage of participants who self-reported their level of knowledge regarding the respective treatment options. Only 17% of those who chose alternative chemotherapy regarded themselves as having a good understanding, whereas 50% of those who opted for PC self-attributed a higher level of knowledge in this area. Additionally, in the other scenario of chemotherapy, a higher percentage of participants (86%) who chose withholding/withdrawal options claimed to have attended both PC and/or EOL classes and law or ethics classes on PC and/or EOL within a span of two years than the group who opted for the full code treatment approach (61%). These findings shed light on the distinct profiles of each group.

In this study, all 20 participants said that they would discuss the care pathway with the patient and multidisciplinary team, which is not commonly seen in daily oncohematology practice in several healthcare services. Moreover, it is important to highlight that our sample comprised a substantial proportion of participants with prior theoretical or practical exposure to PC and/or EOL care. This observation is contrary to the existing literature and highlights the uniqueness of our study population.^8,26,27^ Furthermore, our study included a sample in which 100% of the participants expressed interest in engaging in discussions regarding PC and/or EOL. This high level of interest may be attributed to the fact that our study was conducted in a hospital setting that boasts of robust PC services. This service encompasses a multidisciplinary team that actively involves two palliative doctors in patient discussions within the Hematology Division.

A standout observation in our study was the presence of contrasting responses among nearly 50% of the participants when asked about the most likely course of action and approach they believed to be optimal for the patient. It is crucial to delve deeper into the underlying reasons for such discrepancies, as medical decisions should ideally be guided by evidence-based medicine and established protocols, rather than individual preferences or subjective considerations. This finding raises significant concerns, particularly within a specialized team, where the majority of physicians have received training within the same service and have access to the same patient cohort. Notably, even in the presence of a specialized PC team, the selected approaches varied significantly among nearly 50% of the participants. Considering the broader context of hematologists in cities, states, or countries, it is reasonable to anticipate a greater divergence in clinical practice.

This study has few limitations. First, owing to the cross-sectional design, it was not feasible to establish a causal relationship between the results obtained from the census sample and the observed differences in the percentages and standard deviations. Second, the sample size of 20 respondents is insufficient to provide a reliable estimate for a larger population. This limitation further accounts for the observed differences in percentages, means, and standard deviations. Therefore, it is crucial to evaluate the heterogeneity of hematologists’ practices in PC and EOL care within larger populations to establish potential correlations between physician characteristics and their respective approaches. Also, this study employed single-scenario control within a tertiary hospital that already employed a specialized PC team. Consequently, the generalizability of these findings to other healthcare settings is a challenge. Further investigations involving diverse and representative populations are required to confirm the applicability of these results. In addition, to mitigate potential confounding factors and emphasize the impact of physician characteristics, we deliberately utilized a simplified case scenario, excluding the pivotal roles that patients, families, and surrogates may play in shaping the provision of care.

The strengths of the study include a remarkable 100% response rate which is comparatively higher than what could be achieved in a multicenter study. This is a pilot study and is significantly important as an early contributor to the initiation and fostering of discussions in the field of oncohematology. Further, the questionnaire underwent meticulous piloting and refinement guided by expert opinions to ensure its robustness and reliability. Moreover, the anonymous nature of the questionnaire deserves special mention as it likely fostered an environment conducive to candid responses, particularly given that this topic remains relatively underrepresented and is not widely disseminated in Brazil or globally.

Nonetheless, there has been relatively limited research investigating the impact of physician characteristics on clinical practice.^7,29,30^ Both personal and professional attributes, such as sex, years since graduation, work hours, and even religious beliefs, have been linked to disparities in EOL decision-making.^32-35^ Research conducted in the oncology field has revealed correlations between physicians’ educational credentials and their performance in providing EOL care to patients in critical stages, although a specific lack of research exists in the context of oncohematology.^7,8,16^ These criteria have emerged as a crucial perspective on the variability of PC and EOL care decisions in hematology.^7,8,16,29^

We found it essential to recognize the potential for discovering and investigating further associations. Such investigations hold immense importance in the existing literature, as they have the potential to identify modifiable factors that can effectively enhance the quality of PC and EOL care within the oncohematology setting. For instance, the identification of ethical and legal considerations in PC or EOL may lead to significant changes in clinical practices, especially when considering the “rollercoaster” nature of evolution of oncohematology, with diseases that present high chances of cure and at the same time strong odds of death or intensive deterioration.^11-13^ These strategic findings can subsequently be implemented within healthcare services, along with the potential influence of sociodemographic factors. Therefore, preliminary studies are crucial to reach these significant milestones. This study can be a benchmark for extensive investigations involving larger sample sizes that better represent a larger physician population, to propose new PC models and more specialized clinical and intra-team approaches.^7,29,30^

## CONCLUSION

Our study highlights marked differences in the perceptions of PC and EOL care among hematology professionals within the same center. These disparities may be driven by personal beliefs, previous educational experience with PC and EOL care, and ethical considerations. Physicians’ personal beliefs and knowledge levels may influence their approaches, although all participants in our study expressed interest in PC and EOL care discussions. The study also demonstrated variations between healthcare professionals’ beliefs and practices and persistent historical tendencies to prioritize aggressive interventions in the oncohematology scenario. Further research is needed to understand how physician characteristics affect the perceptions of PC and EOL care among patients with hematological malignancies, paving the way for improved care practices for their patients.

## References

[1] Elliott E, Watson T, Singh D, Wong C, Lo SS (2021). Outcomes of specialty palliative care interventions for patients with hematologic malignancies: a systematic review. J Pain Symptom Manage..

[2] Moreno-Alonso D, Porta-Sales J, Monforte-Royo C (2018). Palliative care in patients with haematological neoplasms: An integrative systematic review. Palliat Med..

[3] Hui D, Didwaniya N, Vidal M (2014). Quality of end-of-life care in patients with hematologic malignancies: a retrospective cohort study. Cancer..

[4] Howell DA, Shellens R, Roman E (2011). Haematological malignancy: are patients appropriately referred for specialist palliative and hospice care? A systematic review and meta-analysis of published data. Palliat Med..

[5] Kaasa S, Loge JH, Aapro M (2018). Integration of oncology and palliative care: a Lancet Oncology Commission. Lancet Oncol..

[6] Forte DN, Vincent JL, Velasco IT, Park M (2012). Association between education in EOL care and variability in EOL practice: a survey of ICU physicians. Intensive Care Med..

[7] Bradley EH, Cramer LD, Bogardus ST (2002). Physicians’ ratings of their knowledge, attitudes, and end-of-life-care practices. Acad Med..

[8] Wright AA, Zhang B, Ray A (2008). Associations between end-of-life discussions, patient mental health, medical care near death, and caregiver bereavement adjustment. JAMA..

[9] Haun MW, Estel S, Rücker G (2017). Early palliative care for adults with advanced cancer. Cochrane Database Syst Rev..

[10] Hui D, De La Cruz M, Mori M (2013). Concepts and definitions for “supportive care,” “best supportive care,” “palliative care,” and “hospice care” in the published literature, dictionaries, and textbooks. Support Care Cancer..

[11] Campos CC, Caldas LM, Silva LT (2020). 10-year real-world data on acute myeloid leukemia: The paradigm of a public health center in Brazil. Blood..

[12] Campos CC, Teixeira L, Salvino MA (2020). 10-year real-world data on acute myeloid leukemia: The paradigm of a public health center in Brazil. J Clin Oncol..

[13] Datoguia TS, Velloso EDRP, Helman R (2018). Overall survival of Brazilian acute myeloid leukemia patients according to the European LeukemiaNet prognostic scoring system: a cross-sectional study. Med Oncol..

[14] Shaulov A, Aviv A, Alcalde J, Zimmermann C (2022). Early integration of palliative care for patients with haematological malignancies. Br J Haematol..

[15] Cheng HWB, Lam KO (2021). Supportive and palliative care in hemato-oncology: how best to achieve seamless integration and subspecialty development?. Ann Hematol..

[16] LeBlanc TW, O’Donnell JD, Crowley-Matoka M (2015). Perceptions of palliative care among hematologic malignancy specialists: a mixed-methods study. J Oncol Pract..

[17] Odejide OO, Salas Coronado DY, Watts CD, Wright AA, Abel GA (2014). End-of-life care for blood cancers: a series of focus groups with hematologic oncologists. J Oncol Pract..

[18] Zimmermann C, Swami N, Krzyzanowska M (2014). Early palliative care for patients with advanced cancer: a cluster-randomised controlled trial. Lancet..

[19] Hui D, Hannon BL, Zimmermann C, Bruera E (2018). Improving patient and caregiver outcomes in oncology: Team-based, timely, and targeted palliative care. CA Cancer J Clin..

[20] Siegel MD (2009). End-of-life decision making in the ICU. Clin Chest Med..

[21] Carlet J, Thijs LG, Antonelli M (2004). Challenges in end-of-life care in the ICU. Statement of the 5th International Consensus Conference in Critical Care: Brussels, Belgium, April 2003. Intensive Care Med..

[22] Soares M, Terzi RG, Piva JP (2007). End-of-life care in Brazil. Intensive Care Med..

[23] Broom A, Kirby E, Good P, Wootton J, Adams J (2013). The art of letting go: referral to palliative care and its discontents. Soc Sci Med..

[24] Allen SL, Davis KS, Rousseau PC (2015). Advanced Care Directives: Overcoming the Obstacles. J Grad Med Educ..

[25] Benini F, Fabris M, Pace DS (2011). Awareness, understanding and attitudes of Italians regarding palliative care. Ann Ist Super Sanita..

[26] Manitta VJ, Philip JA, Cole-Sinclair MF (2010). Palliative care and the hemato-oncological patient: can we live together? A review of the literature. J Palliat Med..

[27] Tendas A, Niscola P, Ales M (2009). Disability and physical rehabilitation in patients with advanced hematological malignancies followed in a home care program. Support Care Cancer..

[28] Julious SA (2005). Two-sided confidence intervals for the single proportion: comparison of seven methods by Robert G. Newcombe, Statistics in Medicine 1998 17:857-872. Stat Med..

[29] Hajjaj FM, Salek MS, Basra MK, Finlay AY (2010). Non-clinical influences on clinical decision-making: a major challenge to evidence-based practice. J R Soc Med..

[30] Garland A, Shaman Z, Baron J, Connors AF (2006). Physician-attributable differences in intensive care unit costs: a single-center study. Am J Respir Crit Care Med..

[31] LeBlanc TW (2014). In the sandbox: palliative care and hematologic malignancies. J Community Support Oncol..

[32] Giannini A, Pessina A, Tacchi EM (2003). End-of-life decisions in intensive care units: attitudes of physicians in an Italian urban setting. Intensive Care Med..

[33] Barnato AE, Hsu HE, Bryce CL (2008). Using simulation to isolate physician variation in intensive care unit admission decision making for critically ill elders with end-stage cancer: a pilot feasibility study. Crit Care Med..

[34] Garland A, Connors AF (2007). Physicians’ influence over decisions to forego life support. J Palliat Med..

[35] Sprung CL, Maia P, Bulow HH (2007). The importance of religious affiliation and culture on end-of-life decisions in European intensive care units. Intensive Care Med..

